# Supplementary material for: Microglia modulate stable wakefulness via the thalamic reticular nucleus in mice
Source: Nat Commun. 2021 Jul 30;12:4646. doi: 10.1038/s41467-021-24915-x (PMC8324895; doi:10.1038/s41467-021-24915-x)
Supplement: Supplementary file 1 — Supplementary Information [file 41467_2021_24915_MOESM1_ESM.pdf]

Supplementary Figure 1

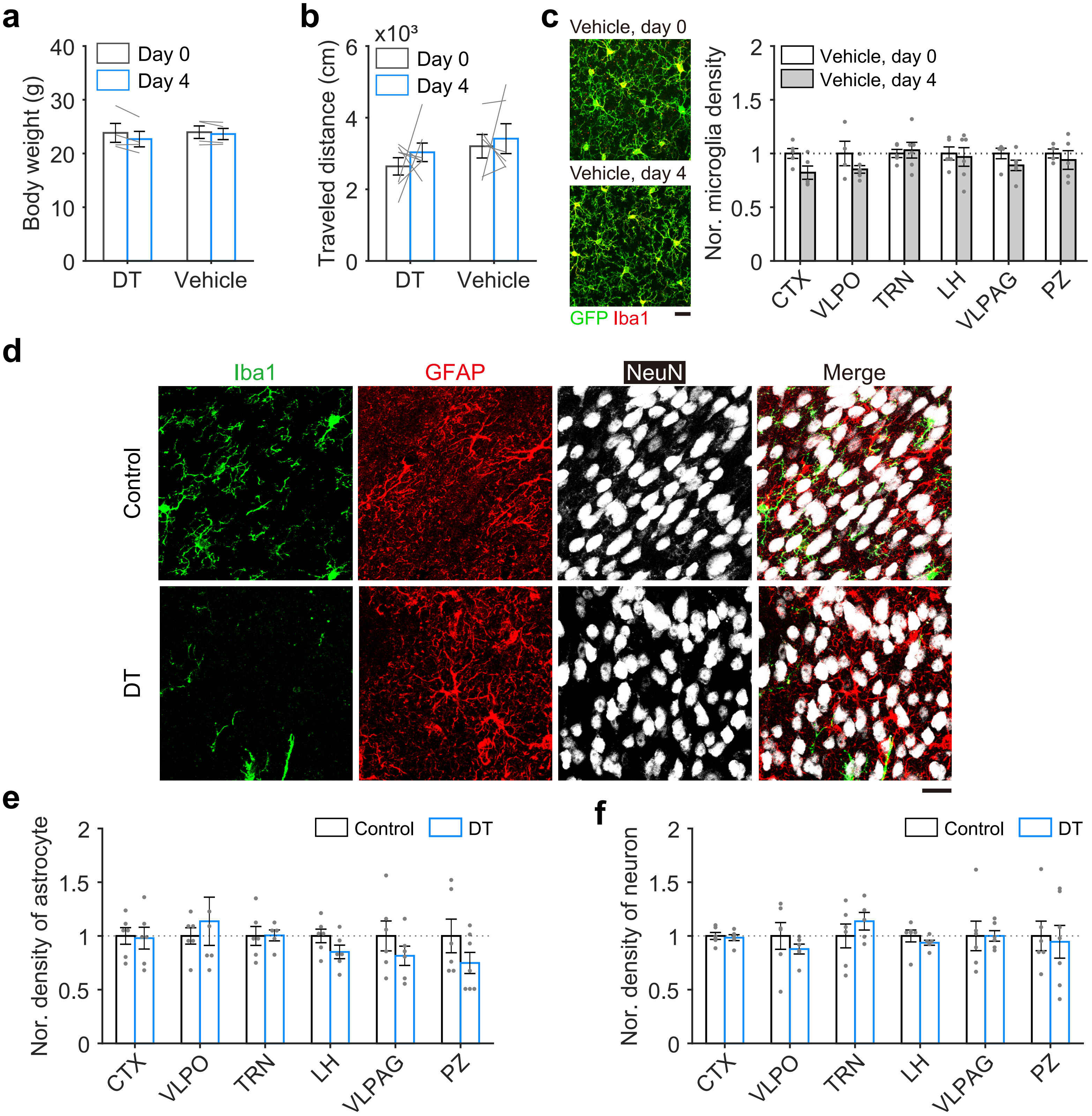

Supplementary Figure 1. Microglial depletion has no effect on body weight, locomotion, and density of astrocyte and neuron in the brain. Related to Figure 1. (**a**) Body weights of mice with DT and vehicle injections (DT, n = 4 mice; Vehicle, n = 4 mice). Each line represents change in body weight from one mouse. (**b)** Total traveled distances for mice with DT and vehicle injections (DT, n = 7 mice; Vehicle, n = 6 mice) during locomotion testing. Each line represents change in locomotion from one mouse. (**c**) Vehicle injection did not affect microglial density across the whole brain. Baseline, n = 5 mice; Vehicle, n = 6 mice. Each dot represents cell density obtained from one mouse. A representative image showing cortical microglia before and after vehicle injection in $CX3CR1^{CerER}$:$R26^{iDTR}$ mice. GFP+/Iba1+ cells represent microglia. CTX, cortex; VLPO, ventrolateral preoptic nucleus; TRN, thalamic reticular nucleus; LH, lateral hypothalamus; VLPAG, ventrolateral periaqueductal gray; PZ, parafacial zone. Scale bar = 50 µm. (**d**) Representative image of microglia, astrocyte and neuron in microglia-depleted mice (DT) and control mice. Scale bar, 20 µm. (**e**) Microglia depletion did not affect astrocyte density across the whole brain. Control, n = 6 images for each examined brain regions from 3 mice; DT, n = 6 images for each examined brain regions from 3 mice. Each dot represents cell density obtained from one image. (**f**) Microglia depletion did not affect neuron density across the whole brain. Control, n = 6 images for each examined brain regions from 3 mice; DT, n = 6 images for each examined brain regions from 3 mice. Each dot represents cell density obtained from one image. $*p < 0.05$; two-sided paired t-test for **a** & **b**, two-sided unpaired t-test for **c**, **e** and **f**. Data are reported as mean ± SEM.

Supplementary Figure 2

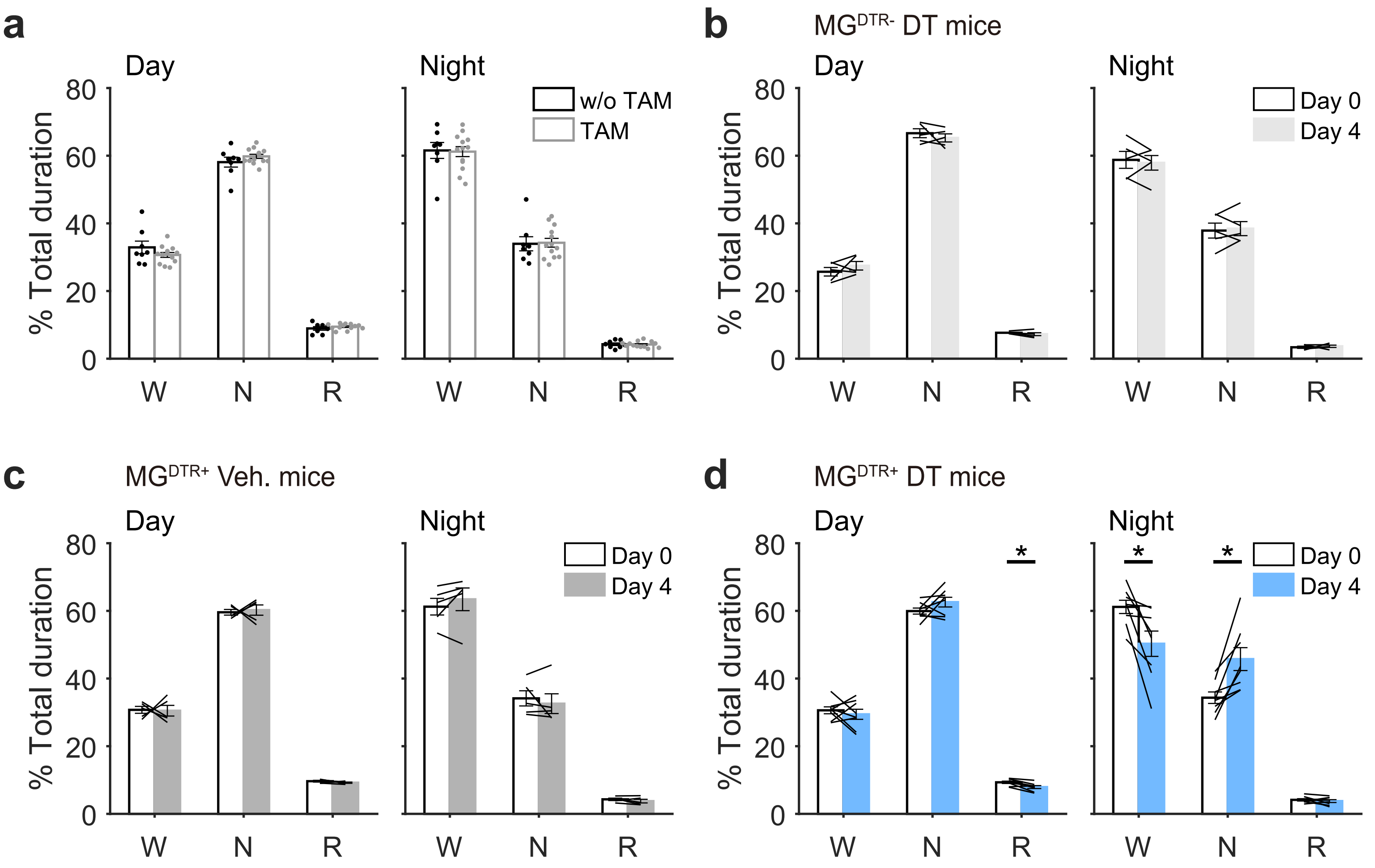

Supplementary Figure 2. Neither tamoxifen nor DT/vehicle treatment *per se* affect global sleep architecture. Related to Figure 1. (**a-c**) Tamoxifen treatment (**a**; w/o TAM, control mice without tamoxifen treatment, 8 mice; TAM, mice with tamoxifen treatment, 13 mice), DT injection (**b**; Day 0, before DT injection; Day 4, after DT injection. 5 mice) and i.p. injection *per se* (**c**; Day 0, before vehicle injection; Day 4, after vehicle injection. 5 mice) have no effect on % total duration for each brain state at both day and night. (**d**) Microglial depletion decreased wakefulness and increased NREM sleep at night (Day 0, before DT injection; Day 4, after DT injection. $p = 0.013$ for wake state, $p = 0.005$ for NREM sleep. 8 mice). Although relative to day 0, REMs was reduced at day 4 in microglia-depleted mice ($p = 0.004$), there was no significant change in difference of % REMs (Day 4 – Day 0) between microglia-depleted mice (MG^{DTR+} DT) and controls (MG^{DTR-} DT and MG^{DTR+} Veh.; **Fig. 1d**), indicating sleep architecture was specifically affected at night following microglial depletion. Each line represents change in % total duration from one animal. *$p < 0.05$; two-sided unpaired t-test for **a**, two-sided paired t-test for **b**-**d**. Data are reported as mean ± SEM.

Supplementary Figure 3

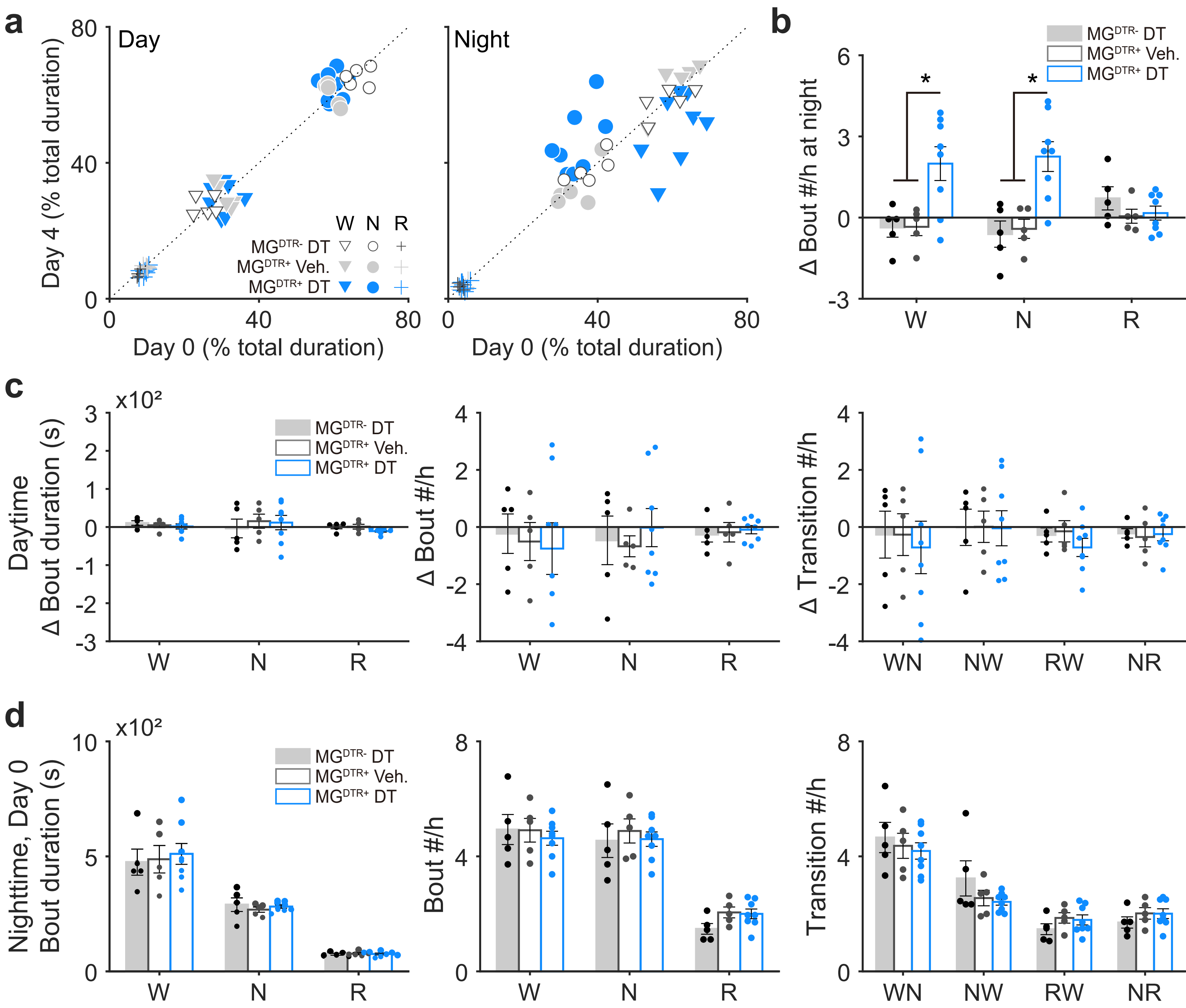

Supplementary Figure 3. Microglia depletion specifically altered sleep architecture at night. Related to Figure 1. (**a**) Scatter plots represent the proportion of each brain state before (Day 0, x-axis) and after (Day 4, y-axis) DT or vehicle injections. W, wakeful (downward-pointing triangle); N, NREM sleep (circle); R, REM sleep (plus sign). (**b**) During nighttime recordings, microglial depletion enhanced the number of both wakeful and NREM sleep bouts. Each dot represents the difference in one mouse before and after microglial depletion (Day 4 – Day 0). The difference was further compared between 3 groups of mice. W: $p = 0.007$ for MG^{DTR-} DT vs MG^{DTR+} DT; $p = 0.008$ for MG^{DTR+} Veh. vs MG^{DTR+} DT; N: $p = 0.001$ for MG^{DTR-} DT vs MG^{DTR+} DT, $p = 0.002$ for MG^{DTR+} Veh. vs MG^{DTR+} DT. MG^{DTR+} DT, n = 8 mice; MG^{DTR+} Veh., n = 5 mice. MG^{DTR-} DT, n = 5 mice. (**c**) During daytime recordings, microglial depletion had no effect on bout duration (left), bout number (middle), or transition number (right). Each dot represents the difference in one mouse before and after microglial depletion (Day 4 – Day 0). The difference was further compared between 3 groups of mice. MG^{DTR+} DT, n = 8 mice; MG^{DTR+} Veh., n = 5 mice. MG^{DTR-} DT, n = 5 mice. (**d**) Mice with and without DTR expression exhibited comparable sleep architectures in terms of bout duration (left), bout number (middle) and transition number (right) before receiving injections. MG^{DTR-} DT, n = 5 mice; MG^{DTR+} Veh., n = 5 mice; MG^{DTR+} DT, n = 8 mice. Each dot represents data from one animal. *$p < 0.05$; one-way ANOVA with Fisher's *post hoc* test for **b**-**d**. Data are reported as mean ± SEM.

Supplementary Figure 4

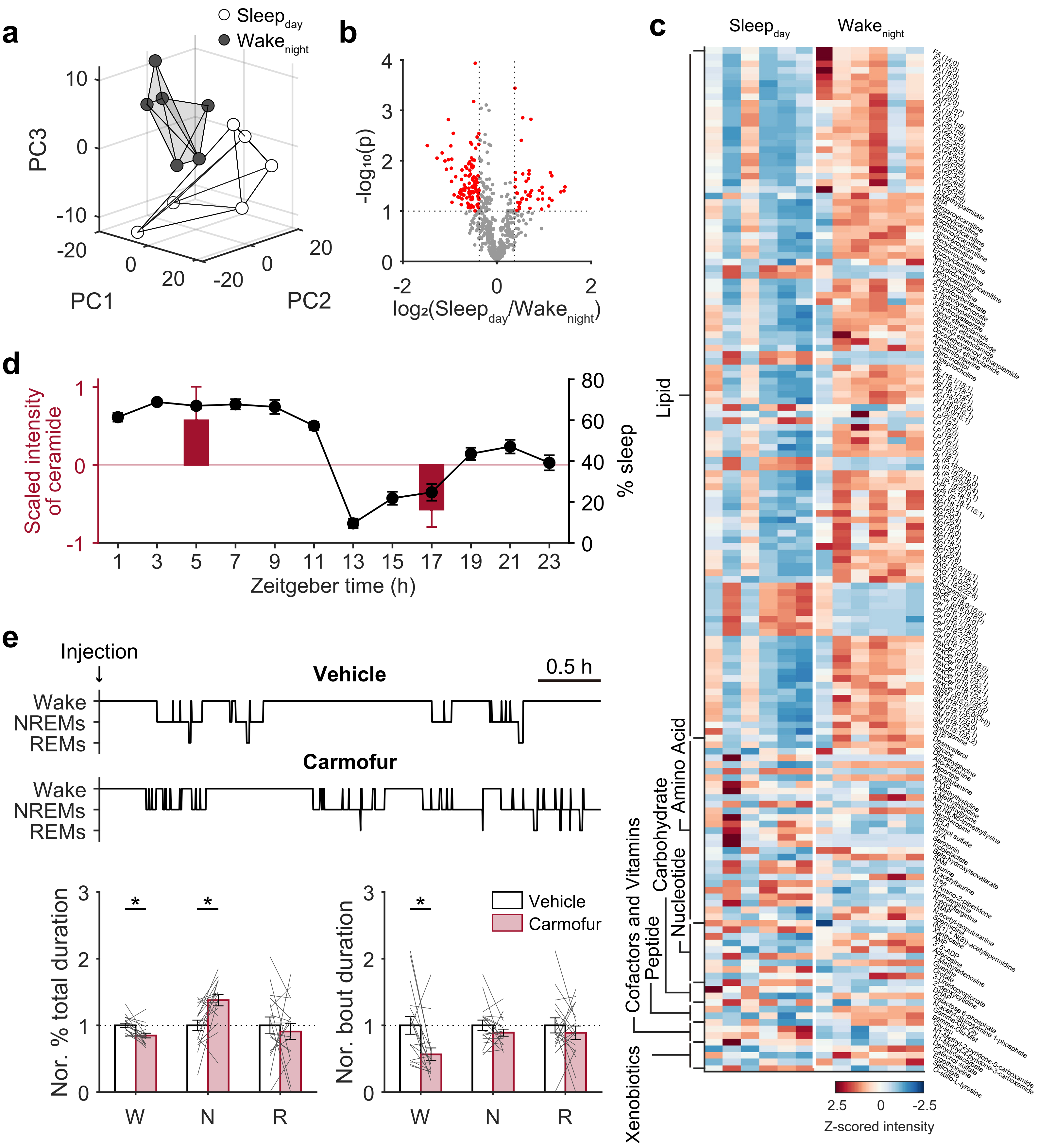

Supplementary Figure 4. Ceramide levels actively modulated sleep/wakefulness behavior. Related to Figure 2. (**a**) Principal component analysis of all metabolites identified in subcortical brain regions. There was a clear separation in metabolite expression between $Sleep_{day}$ and $Wake_{night}$. (**b**) A volcano plot of differentially expressed metabolites between day and night. Red dots represent metabolites with > 1.3 fold-changes between day and night and $p < 0.1$ (two-sided unpaired t-test); these metabolites were included in further analyses. No adjustment was been performed; p-value of differentially expressed metabolites was provided in Supplementary Table 1. (**c**) A heatmap of differentially expressed metabolites between $Sleep_{day}$ and $Wake_{night}$. Each column represents the data from one mouse, and each row represents one metabolite named on the right side. The white line represents the border between the two groups (left, tissue collected during the day, n = 6 mice; right, tissue collected at night, n = 6 mice). The color scale indicates z-score intensity. (**d**) Change in total duration of sleep and subcortical ceramide level between day and night. Mice with EEG/EMG recording, n = 8; Mice for ceramide measurement: day, 8 mice; night, 7 mice. (**e**) An increase in nocturnal ceramide with an acidic ceramidase inhibitor (carmofur) facilitated NREM sleep, and decreased wakefulness, but did not change sleep-bout duration. The top hypnogram is a representative recording from one mouse. n = 17 pairs of recordings from 6 mice. Each line represents a recording from one animal with carmofur/vehicle injections. Nor. % total duration: $p = 0.003$ for W, $p = 0.002$ for N; Nor. Bout duration: $p = 0.011$ for W. $^*p < 0.05$; two-sided paired t-test for **e**. Data are reported as mean ± SEM.

Supplementary Figure 5

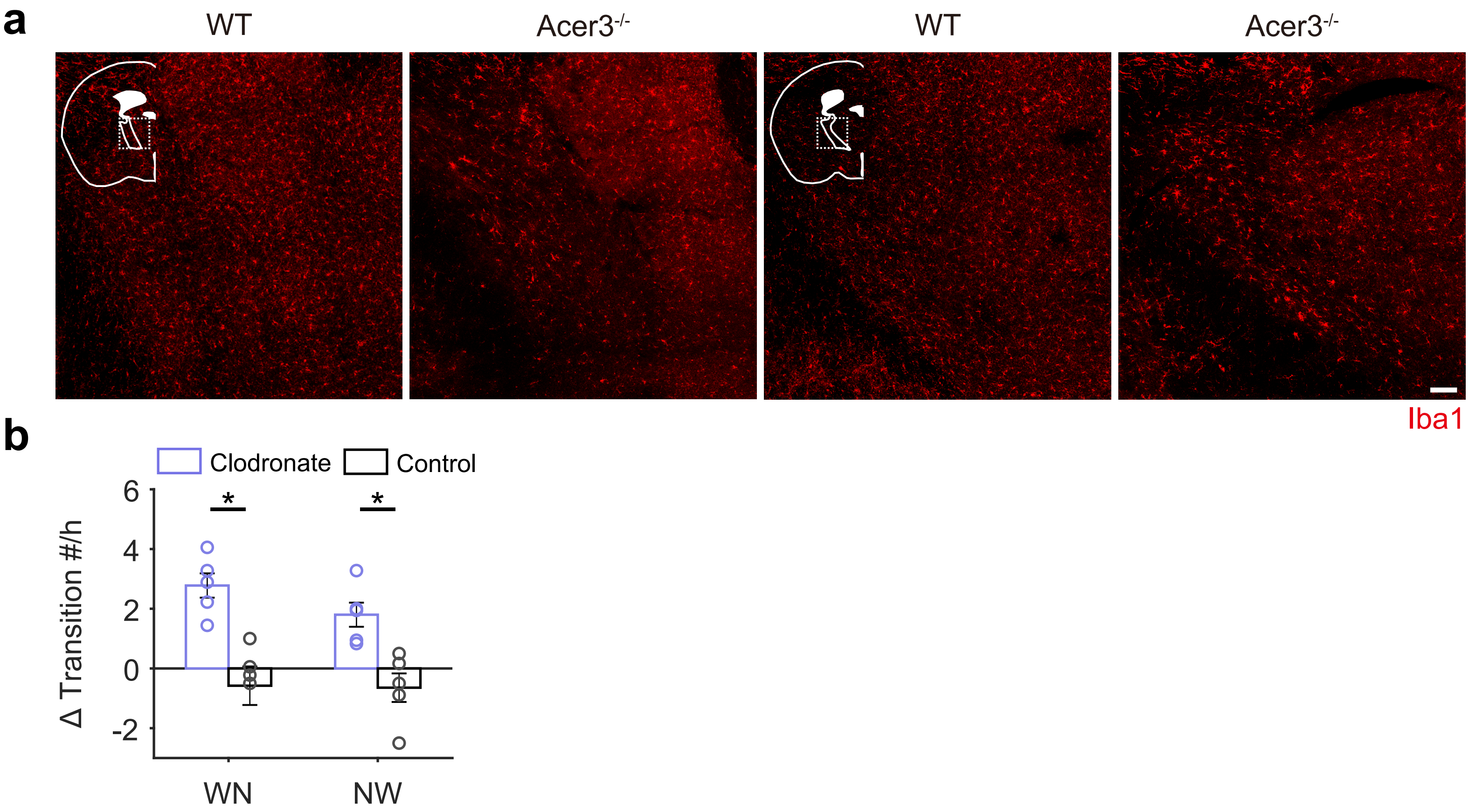

Supplementary Figure 5. aTRN microglia and neurons are sensitive to ceramide concentration. Related to Figure 3. (**a**) Representative images of TRN-containing brain sections with Iba1 staining in wild-type (WT) and Acer3$^{-/-}$ mice. The dashed square in the drawing inset indicates brain regions where images were taken. Scale bar, 100 µm. (**b**) Local depletion of microglia in the aTRN enhanced state transitions between wakefulness and NREM sleep. Clodronate vs Control: $p = 0.004$ for WN; $p = 0.008$ for NW; Clodronate liposomes, n = 5 mice; control liposomes, n = 5 mice. Each dot represents the difference in one mouse before and after clodronate/vehicle injection. *$p < 0.05$; two-sided unpaired t-test for **b** and **c**. Data are reported as mean ± SEM.

Supplementary Figure 6

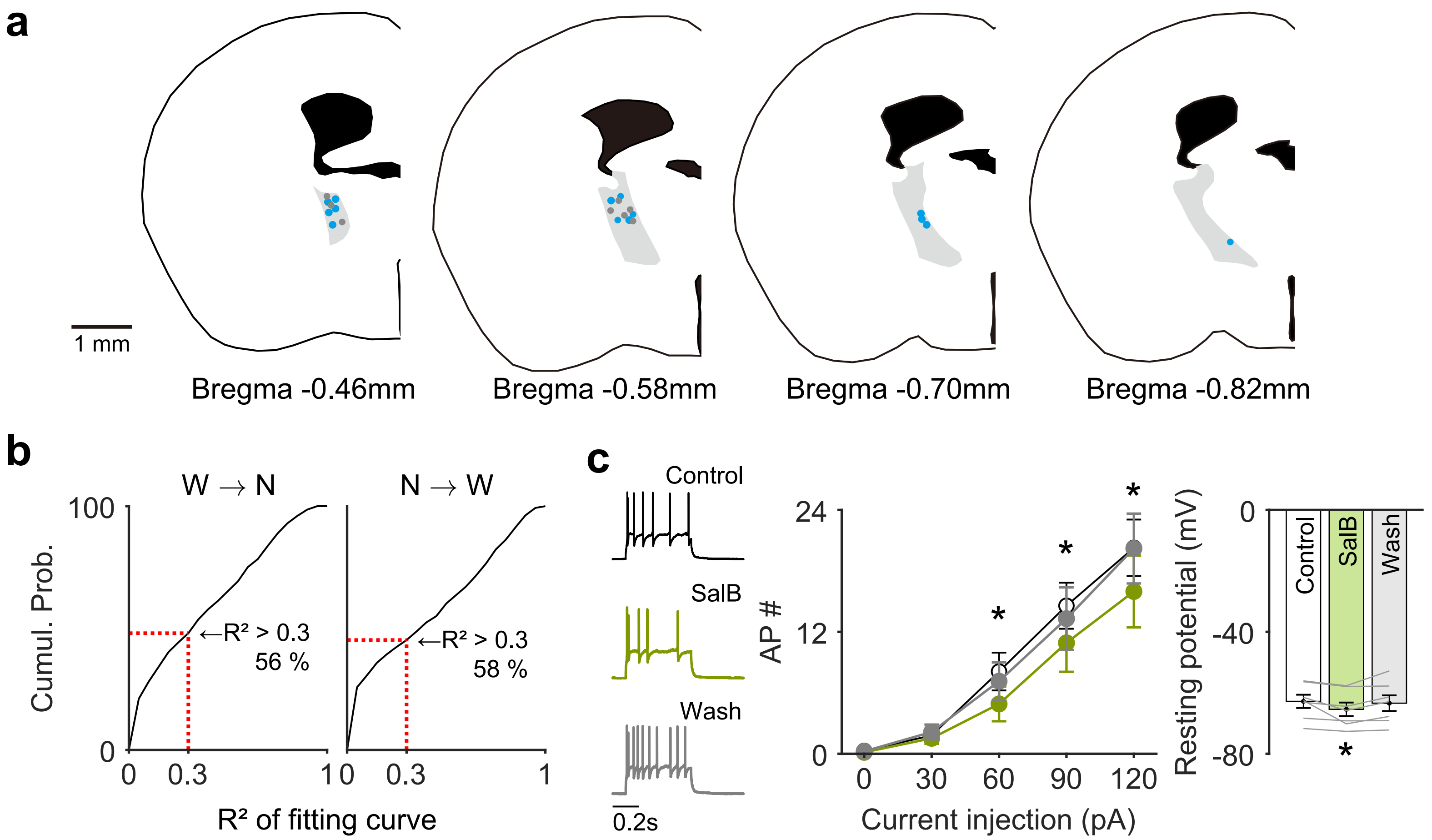

Supplementary Figure 6. Validation of tetrode recording in aTRN and KORD-mediated chemogenetic inhibition of aTRN neuron. Related to Figures 5. (**a**) Recording sites in mice with tetrode implantations in aTRN. The TRN is indicated as the light grey region. Each dot represents the position of a tetrode tip in one mouse, blue dots represent mice used for microglial depletion experiments. Scale bar = 1 mm. (**b**) aTRN neuronal activity was synchronized to transition onsets between wakefulness and NREM sleep. Neuronal activity was fit with a logistic function; R^2 >0.3 was considered to be a good fit. Activity from 56% of TRN neurons during transitions from wakefulness to NREM sleep and from 58% of TRN neurons during transitions from NREM sleep to wakefulness were well fit by the logistic function. (**c**) SalB effectively inhibited KORD-expressing GAD-positive aTRN neurons. SalB infusion attenuated neuronal responses to current injection (left, n = 7 cells) and hyperpolarized the membrane potential (right, n = 7 cells, p = 0.033 for Control vs SalB). Each line in right panel represents resting potential obtained from one cell. *p < 0.05; two-sided paired t-test for **c**. Data are reported as mean ± SEM.

Supplementary Figure 7

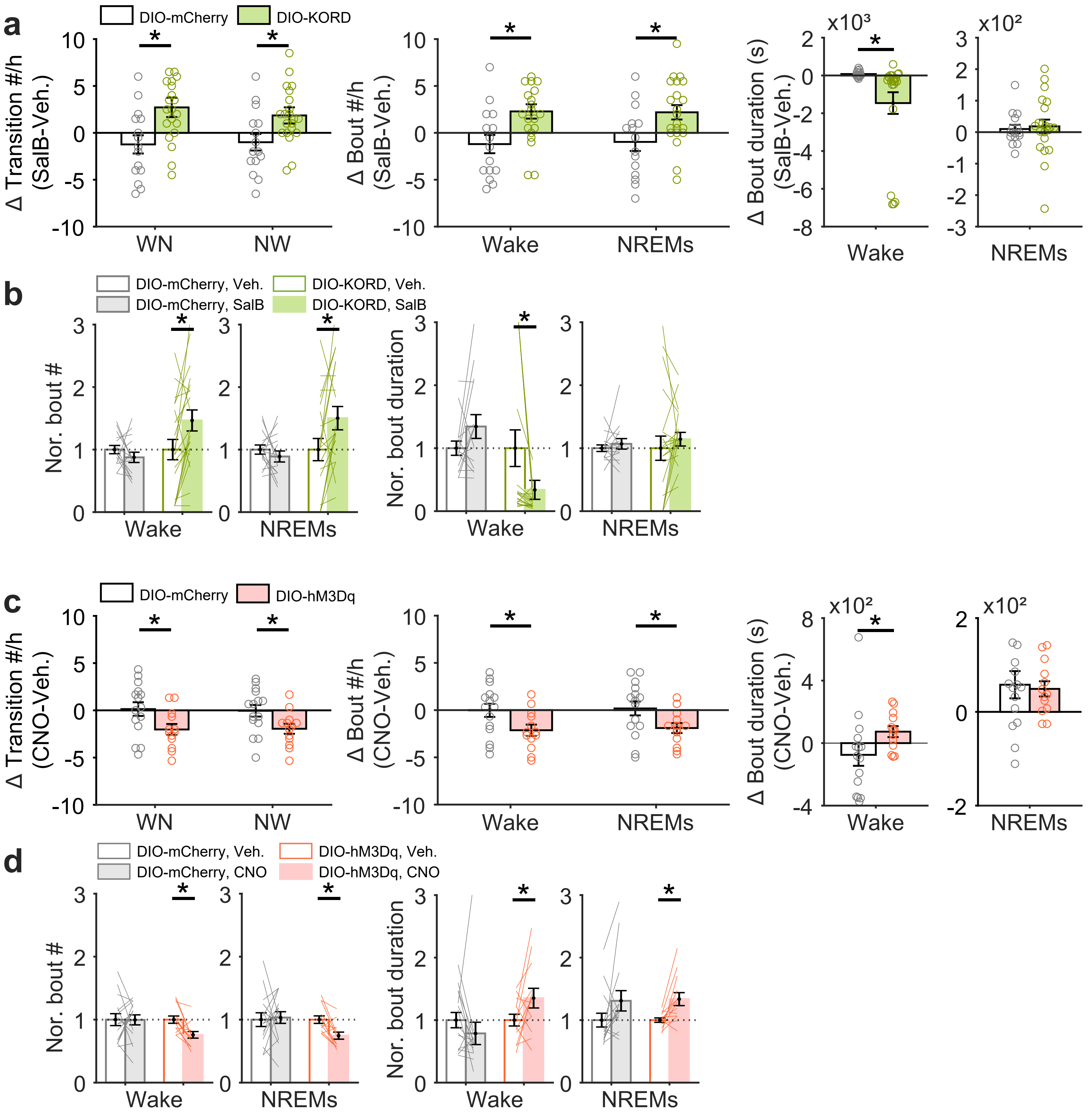

Supplementary Figure 7. Chemogenetic manipulation of aTRN neuronal activity alters sleep architecture. Related to Figures 5. (**a**-**b**) Chemogenetic silencing of KORD-expressing aTRN neurons increased the transition between wake and NREM sleep (**a**, left; DIO-mCherry vs DIO-KORD, $p = 0.006$ for WN, $p = 0.016$ for NW), the number of wakeful and NREM sleep bouts (**a**, middle; DIO-mCherry vs DIO-KORD, $p = 0.012$ for Wake, $p = 0.031$ for NREM sleep. **b**, left; DIO-KORD, Veh vs SalB: $p = 0.007$ for wake; $p = 0.010$ for NREM sleep), and decreased the bout duration of wake (**a**, right; DIO-mCherry vs DIO-KORD, $p = 6x10^{-4}$ for Wake. **b**, right; DIO-KORD, Veh vs SalB: $p = 0.019$ for wake; $p = 0.010$ for NREM sleep), but had no impact on sleep-bout duration (**a**, right). DIO-KORD, 21 pairs of recordings collected from 7 mice; DIO-mCherry, 15 pairs of recordings collected from 5 mice. Each open circle in **a** represents the difference in one mouse between SalB/vehicle injections, and then differences were compared in mice with DIO-KORD/DIO-mCherry expression. Each line in **b** represents a recording from one animal with SalB/vehicle injections. (**c**-**d**) Chemogenetic activation of hM3Dq-expressing aTRN neurons decreased the transition between wake and NREM sleep (**c**, left; DIO-mCherry vs DIO-hM3Dq, $p = 0.028$ for WN, $p = 0.019$ for NW), the number of wakeful and NREM sleep bouts (**c**, middle; DIO-mCherry vs DIO-hM3Dq, $p = 0.038$ for Wake, $p = 0.04$ for NREM sleep; **d**, left; DIO- hM3Dq, Veh vs CNO: $p = 0.003$ for wake; $p = 0.002$ for NREM sleep), and increased the bout duration of wake (**c**, right; DIO-mCherry vs DIO-hM3Dq, $p = 0.021$ for Wake; **d**, right; DIO- hM3Dq, Veh vs CNO: $p = 0.0498$ for wake), but had no effect on sleep-bout duration (**d**, right). DIO-hM3Dq, 13 pairs of recordings collected from 4 mice; DIO-mCherry, 15 pairs of recordings collected from 5 mice. Each open circle in **c** represents the difference in one mouse between CNO/vehicle injections, and then differences were compared in mice with DIO-hM3Dq/DIO-mCherry expression. Each line in **d** represents a recording from one animal with CNO/vehicle injections. Note that CNO injection slightly extended bout duration of NREM sleep in mice with DIO-hM3Dq or DIO-mCherry expression (**d**, right; Veh. vs CNO: $p = 0.006$ for DIO-hM3Dq; $p = 0.066$ for DIO-mCherry), while the difference between vehicle and CNO injection was comparable in two groups of mice (**c**, right), indicating the observed effect on bout duration of NREM sleep is induced by CNO *per se*, rather activation of aTRN neurons. $*p < 0.05$; two-sided paired Wilcoxon signed rank test **b** and **d**, two-sided Mann-Whitney test for **a** and **c**. Data are reported as mean ± SEM.

Supplementary Figure 8

a

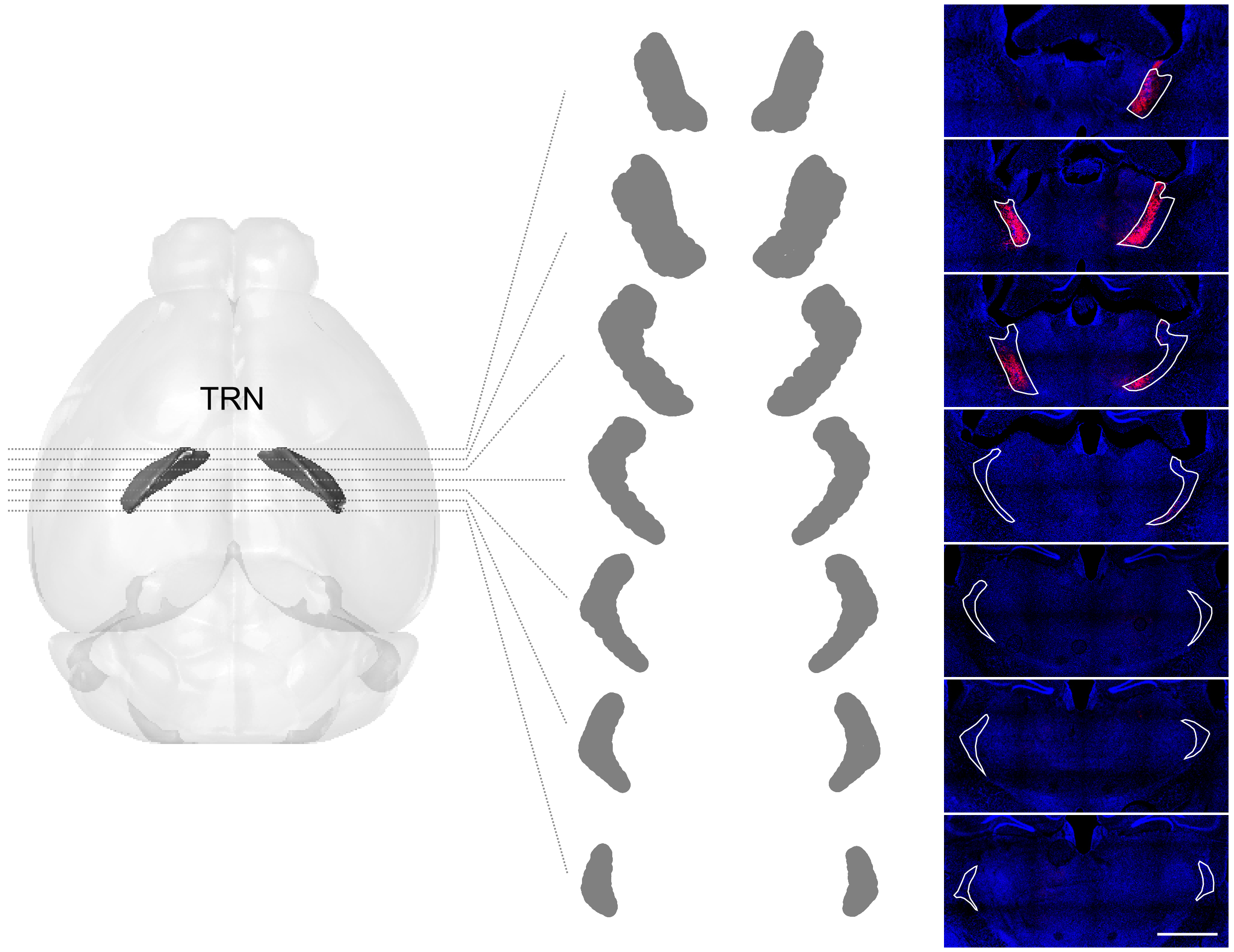

b

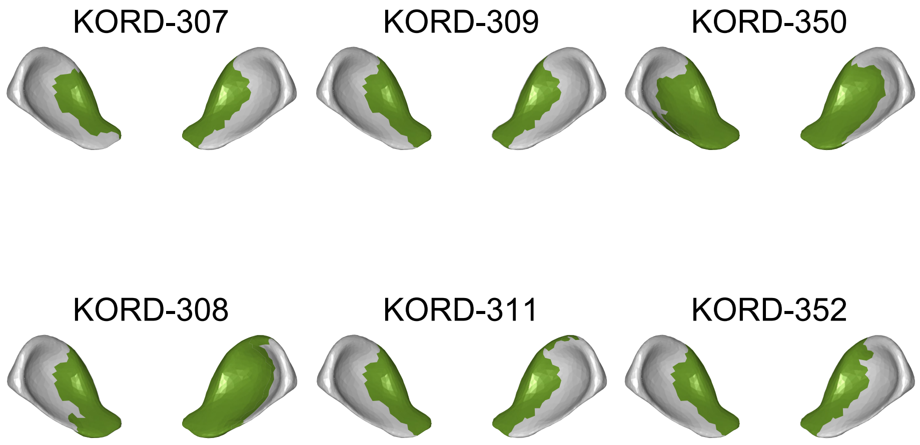

c

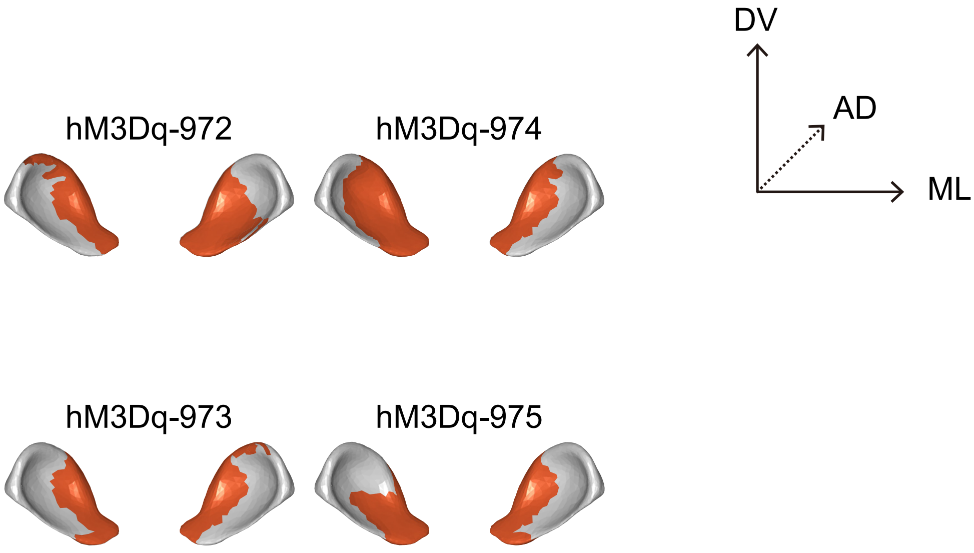

Supplementary Figure 8. Validation of virus spreading in the aTRN. Related to Figure 5. (**a**) Validation of the range of virus spread in the TRN. We evenly divided the whole TRN into seven parts from anterior to posterior (left and middle). Whole-brain coronal images from virus-expressing mice were collected (right), and their virus spread ranges were determined based on observations of infected TRN neuronal cell bodies, and all images were aligned from anterior to posterior based on the cartoon drawing shown in the middle. The whole TRN was reconstructed, and colored areas represent TRN regions with virus expression. Representative images were collected from animal 975, images from other mice were schematically shown in b & c. Scale bar = 1 mm. (**b**) Expression of cre-dependent KORD was mainly restricted to the aTRN (n = 6 mice, green) in GAD2cre mice. Although virus expression in animal 308 infected few neurons in one side of the posterior TRN, we did not observe any difference in sleep architecture relative to other animals. AP, anterior-posterior axis; ML, medial-lateral axis; DV; dorsal-ventral axis. (**c**) Expression of cre-dependent hM3Dq was mainly restricted to the aTRN (n = 4 mice, red) in GAD2cre mice. Although virus expression in animal 974 affected few neurons in one side of the posterior TRN, we did not observe any difference in sleep architecture relative to other animals.

Supplementary Figure 9

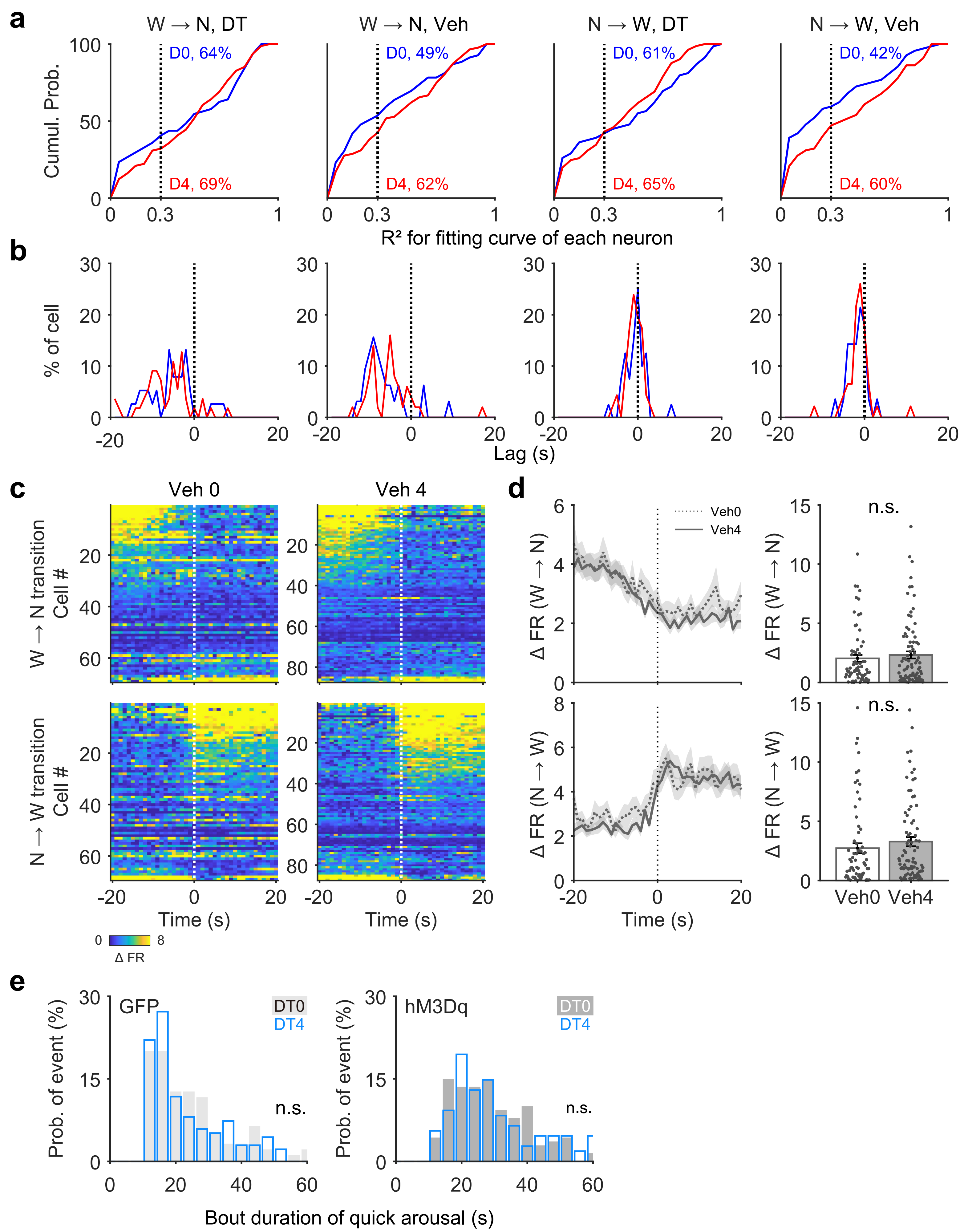

Supplementary Figure 9. Rescue of attenuated aTRN neuronal activity restores stable wakefulness in microglia-depleted mice. Related to Figure 6. (**a**) Neither DT nor vehicle injections had an effect on the proportion of neurons fit by the logistic function. Neurons with $R^2 > 0.3$ were considered a good fit to the logistic function. D0, Day 0; D4, Day 4. (**b**) Neither DT nor vehicle injections had an effect on aTRN neuron lag-times relative to transition onsets. (**c**) A heatmap of nighttime aTRN neuronal activity during transitions from wakefulness to NREM sleep (top, W→N) and from NREM sleep to wakefulness (bottom, N→W) before (left, Veh 0) and after (right, Veh 4) vehicle injections. Each row represents one neuron. Colors represent changes in firing rates during transition-state periods. The activity of each neuron was normalized by its minimum firing rate during the transition period. The averaged activity of aTRN neurons 20 s before and after the onset transitions from wakefulness to NREM sleep (top left) and from NREM sleep to wakefulness (bottom right) was comparable before and after vehicle injections. V0, n = 69 neurons from 6 mice; V4, n = 87 neurons from 6 mice. (**d**) Statistical analysis of firing rate differences between wakefulness and NREM sleep during transition periods (W→N, V0, n = 69 neurons, V4, n = 87 neurons, 6 mice; N→W, V0, n = 69 neurons, V4, n = 87 neurons, 6 mice). Spike events that occurred within 5–20 s before and after transition onsets were included for each neuron. The results further confirmed that aTRN neuronal firing in mice with vehicle injections remained intact. (**e**) Microglia depletion had no effects on quick arousal in mice with chemogenetic activation of the aTRN (left panel, n = 7 mice) and in mice with GFP-only expression (right panel, n = 7 mice). *$p < 0.05$; two-sided unpaired t-test for **d**, two-sided Kolmogorov-Smirnov test for **e**. Data are reported as mean ± SEM.

Supplementary Table 1. Differential levels of metabolites between daytime and nighttime in mouse subcortical regions. P value was calculated with two-sided unpaired t-test, no adjustment was performed. Related to Figure 2.

BIOCHEMICAL	SUPER_PATHWAY	SUB_PATHWAY	Fold Change (Day/Night)	P value
glycine	Amino Acid	Glycine, Serine and Threonine Metabolism	0.64	0.05
dimethylglycine	Amino Acid	Glycine, Serine and Threonine Metabolism	0.71	0.01
allo-threonine	Amino Acid	Glycine, Serine and Threonine Metabolism	1.46	0.06
aspartate	Amino Acid	Alanine and Aspartate Metabolism	0.75	0.03
pyroglutamine*	Amino Acid	Glutamate Metabolism	1.64	0.04
N-acetyl-aspartyl-glutamate (NAAG)	Amino Acid	Glutamate Metabolism	0.70	0.04
1-methylhistidine	Amino Acid	Histidine Metabolism	2.20	0.02
3-methylhistidine	Amino Acid	Histidine Metabolism	2.24	0.02
N6-methyllysine	Amino Acid	Lysine Metabolism	0.70	0.02
N6,N6,N6-trimethyllysine	Amino Acid	Lysine Metabolism	1.44	0.06
saccharopine	Amino Acid	Lysine Metabolism	0.59	0.05
3-(4-hydroxyphenyl)lactate (HPLA)	Amino Acid	Tyrosine Metabolism	1.36	0.07
phenol sulfate	Amino Acid	Tyrosine Metabolism	1.67	0.04
homovanillate (HVA)	Amino Acid	Tyrosine Metabolism	1.93	0.09
serotonin	Amino Acid	Tryptophan Metabolism	1.83	0.06
indolelactate	Amino Acid	Tryptophan Metabolism	1.39	0.09
beta-hydroxyisovalerate	Amino Acid	Leucine, Isoleucine and Valine Metabolism	0.70	0.06
S-adenosylmethionine (SAM)	Amino Acid	Methionine, Cysteine, SAM and Taurine Metabolism	0.71	0.00
taurine	Amino Acid	Methionine, Cysteine, SAM and Taurine Metabolism	1.52	0.04
N-acetyltaurine	Amino Acid	Methionine, Cysteine, SAM and Taurine Metabolism	1.86	0.04
urea	Amino Acid	Urea cycle; Arginine and Proline Metabolism	0.69	0.00
3-amino-2-piperidone	Amino Acid	Urea cycle; Arginine and Proline Metabolism	2.14	0.08
homoarginine	Amino Acid	Urea cycle; Arginine and Proline Metabolism	1.43	0.00
N-acetylarginine	Amino Acid	Urea cycle; Arginine and Proline Metabolism	1.94	0.03
N,N,N-trimethyl-alanylproline betaine (TMAP)	Amino Acid	Urea cycle; Arginine and Proline Metabolism	1.40	0.07

N-acetyl-isoputreanine	Amino Acid	Polyamine Metabolism	0.76	0.07
spermidine	Amino Acid	Polyamine Metabolism	0.71	0.03
(N(1) + N(8))-acetylspermidine	Amino Acid	Polyamine Metabolism	1.46	0.00
gamma-glutamylglycine	Peptide	Gamma-glutamyl Amino Acid	0.56	0.05
gamma-glutamylmethionine	Peptide	Gamma-glutamyl Amino Acid	0.73	0.01
glycylleucine	Peptide	Dipeptide	0.75	0.08
dihydroxyacetone phosphate (DHAP)	Carbohydrate	Glycolysis, Gluconeogenesis, and Pyruvate Metabolism	1.40	0.07
galactose 6-phosphate	Carbohydrate	Fructose, Mannose and Galactose Metabolism	0.66	0.04
N-acetyl-glucosamine 1-phosphate	Carbohydrate	Aminosugar Metabolism	1.31	0.02
myristate (14:0)	Lipid	Long Chain Saturated Fatty Acid	0.77	0.06
pentadecanoate (15:0)	Lipid	Long Chain Saturated Fatty Acid	0.72	0.03
palmitate (16:0)	Lipid	Long Chain Saturated Fatty Acid	0.71	0.03
margarate (17:0)	Lipid	Long Chain Saturated Fatty Acid	0.73	0.03
stearate (18:0)	Lipid	Long Chain Saturated Fatty Acid	0.68	0.01
nonadecanoate (19:0)	Lipid	Long Chain Saturated Fatty Acid	0.72	0.01
arachidate (20:0)	Lipid	Long Chain Saturated Fatty Acid	0.69	0.01
10-heptadecenoate (17:1n7)	Lipid	Long Chain Monounsaturated Fatty Acid	0.68	0.00
oleate/vaccenate (18:1)	Lipid	Long Chain Monounsaturated Fatty Acid	0.71	0.08
10-nonadecenoate (19:1n9)	Lipid	Long Chain Monounsaturated Fatty Acid	0.68	0.04
eicosenoate (20:1n9 or 1n11)	Lipid	Long Chain Monounsaturated Fatty Acid	0.66	0.08
erucate (22:1n9)	Lipid	Long Chain Monounsaturated Fatty Acid	0.66	0.08
docosapentaenoate (DPA; 22:5n3)	Lipid	Long Chain Polyunsaturated Fatty Acid (n3 and n6)	0.64	0.01
docosahexaenoate (DHA; 22:6n3)	Lipid	Long Chain Polyunsaturated Fatty Acid (n3 and n6)	0.63	0.01
nisinate (24:6n3)	Lipid	Long Chain Polyunsaturated Fatty Acid (n3 and n6)	0.59	0.04
linoleate (18:2n6)	Lipid	Long Chain Polyunsaturated Fatty Acid (n3 and n6)	0.71	0.02
dihomolinoleate (20:2n6)	Lipid	Long Chain Polyunsaturated Fatty Acid (n3 and n6)	0.69	0.06
dihomolinolenate (20:3n3 or 3n6)	Lipid	Long Chain Polyunsaturated Fatty Acid (n3 and n6)	0.66	0.04
adrenate (22:4n6)	Lipid	Long Chain Polyunsaturated Fatty Acid (n3 and n6)	0.72	0.10
docosadienoate (22:2n6)	Lipid	Long Chain Polyunsaturated Fatty Acid (n3 and n6)	0.66	0.07

mead acid (20:3n9)	Lipid	Long Chain Polyunsaturated Fatty Acid (n3 and n6)	0.68	0.04
(14 or 15)-methylpalmitate (a17:0 or i17:0)	Lipid	Fatty Acid, Branched	0.70	0.05
methylmalonate (MMA)	Lipid	Fatty Acid Metabolism (also BCAA Metabolism)	0.52	0.07
margaroylcarnitine (C17)*	Lipid	Fatty Acid Metabolism (Acyl Carnitine, Long Chain Saturated)	0.67	0.01
stearoylcarnitine (C18)	Lipid	Fatty Acid Metabolism (Acyl Carnitine, Long Chain Saturated)	0.71	0.02
arachidoylcarnitine (C20)*	Lipid	Fatty Acid Metabolism (Acyl Carnitine, Long Chain Saturated)	0.61	0.05
behenoylcarnitine (C22)*	Lipid	Fatty Acid Metabolism (Acyl Carnitine, Long Chain Saturated)	0.59	0.04
lignoceroylcarnitine (C24)*	Lipid	Fatty Acid Metabolism (Acyl Carnitine, Long Chain Saturated)	0.63	0.03
oleoylcarnitine (C18:1)	Lipid	Fatty Acid Metabolism (Acyl Carnitine, Monounsaturated)	0.75	0.00
eicosenoylcarnitine (C20:1)*	Lipid	Fatty Acid Metabolism (Acyl Carnitine, Monounsaturated)	0.63	0.02
erucoylcarnitine (C22:1)*	Lipid	Fatty Acid Metabolism (Acyl Carnitine, Monounsaturated)	0.60	0.06
nervonoylcarnitine (C24:1)*	Lipid	Fatty Acid Metabolism (Acyl Carnitine, Monounsaturated)	0.51	0.03
(S)-3-hydroxybutyrylcarnitine	Lipid	Fatty Acid Metabolism (Acyl Carnitine, Hydroxy)	0.70	0.04
deoxycarnitine	Lipid	Carnitine Metabolism	1.63	0.05
palmitoylcholine	Lipid	Fatty Acid Metabolism (Acyl Choline)	1.58	0.05
2-hydroxybehenate	Lipid	Fatty Acid, Monohydroxy	0.51	0.01
2-hydroxynervonate*	Lipid	Fatty Acid, Monohydroxy	0.49	0.01
3-hydroxypalmitate	Lipid	Fatty Acid, Monohydroxy	0.66	0.01
3-hydroxystearate	Lipid	Fatty Acid, Monohydroxy	0.67	0.04
oleoyl ethanolamide	Lipid	Endocannabinoid	0.77	0.04
palmitoyl ethanolamide	Lipid	Endocannabinoid	0.76	0.04
stearoyl ethanolamide	Lipid	Endocannabinoid	0.75	0.05
docosahexaenoyl ethanolamide	Lipid	Endocannabinoid	0.76	0.06
arachidoyl ethanolamide (20:0)*	Lipid	Endocannabinoid	0.59	0.07
N-palmitoylserine	Lipid	Endocannabinoid	0.68	0.02
chiro-inositol	Lipid	Inositol Metabolism	0.68	0.09
phosphocholine	Lipid	Phospholipid Metabolism	1.46	0.02
phosphoethanolamine (PE)	Lipid	Phospholipid Metabolism	1.38	0.08
1,2-dioleoyl-GPE (18:1/18:1)	Lipid	Phosphatidylethanolamine (PE)	0.73	0.04

1-oleoyl-2-linoleoyl-GPE (18:1/18:2)*	Lipid	Phosphatidylethanolamine (PE)	0.69	0.08
1,2-dioleoyl-GPS (18:1/18:1)	Lipid	Phosphatidylserine (PS)	0.73	0.02
1,2-dipalmitoyl-GPG (16:0/16:0)	Lipid	Phosphatidylglycerol (PG)	0.67	0.04
1-stearoyl-2-oleoyl-GPG (18:0/18:1)	Lipid	Phosphatidylglycerol (PG)	0.70	0.01
1-palmitoyl-2-oleoyl-GPI (16:0/18:1)*	Lipid	Phosphatidylinositol (PI)	0.69	0.05
1-arachidonoyl-GPC* (20:4)*	Lipid	Lysophospholipid	1.34	0.10
2-stearoyl-GPE (18:0)*	Lipid	Lysophospholipid	0.75	0.08
1-palmitoyl-GPS (16:0)*	Lipid	Lysophospholipid	2.55	0.04
1-oleoyl-GPS (18:1)	Lipid	Lysophospholipid	0.62	0.03
1-palmitoyl-GPG (16:0)*	Lipid	Lysophospholipid	0.69	0.03
1-stearoyl-GPG (18:0)	Lipid	Lysophospholipid	0.67	0.05
1-oleoyl-GPG (18:1)*	Lipid	Lysophospholipid	0.73	0.04
1-(1-enyl-palmitoyl)-2-oleoyl-GPE (P-16:0/18:1)*	Lipid	Plasmalogen	0.75	0.04
1-(1-enyl-palmitoyl)-2-palmitoyl-GPC (P-16:0/16:0)*	Lipid	Plasmalogen	1.54	0.06
1-(1-enyl-palmitoyl)-2-arachidonoyl-GPE (P-16:0/20:4)*	Lipid	Plasmalogen	1.31	0.03
1-(1-enyl-palmitoyl)-2-oleoyl-GPC (P-16:0/18:1)*	Lipid	Plasmalogen	0.76	0.09
1-(1-enyl-oleoyl)-GPE (P-18:1)*	Lipid	Lysoplasmalogen	0.73	0.04
1-(1-enyl-oleoyl)-2-oleoyl-GPE (P-18:1/18:1)*	Lipid	Lysoplasmalogen	0.76	0.04
1-oleoylglycerol (18:1)	Lipid	Monoacylglycerol	0.53	0.05
1-dihomo-linolenylglycerol (20:3)	Lipid	Monoacylglycerol	0.41	0.01
1-arachidonylglycerol (20:4)	Lipid	Monoacylglycerol	0.45	0.01
1-docosahexaenoylglycerol (22:6)	Lipid	Monoacylglycerol	0.51	0.01
2-palmitoylglycerol (16:0)	Lipid	Monoacylglycerol	0.61	0.05
2-oleoylglycerol (18:1)	Lipid	Monoacylglycerol	0.60	0.02
2-linoleoylglycerol (18:2)	Lipid	Monoacylglycerol	0.36	0.01
2-arachidonoylglycerol (20:4)	Lipid	Monoacylglycerol	0.47	0.01
2-docosahexaenoylglycerol (22:6)*	Lipid	Monoacylglycerol	0.54	0.02
palmitoyl-oleoyl-glycerol (16:0/18:1) [2]*	Lipid	Diacylglycerol	0.67	0.02

oleoyl-oleoyl-glycerol (18:1/18:1) [2]*	Lipid	Diacylglycerol	0.59	0.02
stearoyl-arachidonoyl-glycerol (18:0/20:4) [2]*	Lipid	Diacylglycerol	0.76	0.04
stearoyl-docosahexaenoyl-glycerol (18:0/22:6) [2]*	Lipid	Diacylglycerol	0.76	0.06
sphinganine	Lipid	Sphingolipid Synthesis	0.73	0.00
N-palmitoyl-sphinganine (d18:0/16:0)	Lipid	Dihydroceramides	1.96	0.06
N-stearoyl-sphinganine (d18:0/18:0)*	Lipid	Dihydroceramides	2.25	0.06
N-palmitoyl-sphingosine (d18:1/16:0)	Lipid	Ceramides	1.95	0.04
N-stearoyl-sphingosine (d18:1/18:0)*	Lipid	Ceramides	2.17	0.06
N-stearoyl-sphingadienine (d18:2/18:0)*	Lipid	Ceramides	2.68	0.04
N-behenoyl-sphingadienine (d18:2/22:0)*	Lipid	Ceramides	1.34	0.04
ceramide (d18:1/17:0, d17:1/18:0)*	Lipid	Ceramides	2.72	0.03
ceramide (d18:1/20:0, d16:1/22:0, d20:1/18:0)*	Lipid	Ceramides	1.63	0.09
glycosyl-N-stearoyl-sphinganine (d18:0/18:0)*	Lipid	Hexosylceramides (HCER)	0.66	0.02
glycosyl-N-arachidoyl-sphingosine (d18:1/20:0)*	Lipid	Hexosylceramides (HCER)	0.70	0.01
glycosyl-N-erucoyl-sphingosine (d18:1/22:1)*	Lipid	Hexosylceramides (HCER)	0.67	0.03
glycosyl-N-tricosenoyl-sphingosine (d18:1/23:1)*	Lipid	Hexosylceramides (HCER)	0.70	0.04
glycosyl-N-nervonoyl-sphingosine (d18:1/24:1)*	Lipid	Hexosylceramides (HCER)	0.76	0.02
glycosyl ceramide (d18:2/24:1, d18:1/24:2)*	Lipid	Hexosylceramides (HCER)	0.69	0.01
glycosyl ceramide (d18:2/25:1, d18:1/25:2)	Lipid	Hexosylceramides (HCER)	0.74	0.02
behenoyl dihydrosphingomyelin (d18:0/22:0)*	Lipid	Dihydrosphingomyelins	0.58	0.04
hydroxypalmitoyl sphingomyelin (d18:1/16:0(OH))	Lipid	Sphingomyelins	0.70	0.02
behenoyl sphingomyelin (d18:1/22:0)*	Lipid	Sphingomyelins	0.68	0.06
lignoceroyl sphingomyelin (d18:1/24:0)	Lipid	Sphingomyelins	0.61	0.04
sphingomyelin (d18:2/23:0, d18:1/23:1, d17:1/24:1)*	Lipid	Sphingomyelins	0.62	0.04

sphingomyelin (d18:2/24:1, d18:1/24:2)*	Lipid	Sphingomyelins	0.66	0.04
sphingosine	Lipid	Sphingosines	0.77	0.00
sphingosine 1-phosphate	Lipid	Sphingosines	0.66	0.08
desmosterol	Lipid	Sterol	0.75	0.04
xanthosine	Nucleotide	Purine Metabolism, (Hypo)Xanthine/Inosine containing	1.54	0.03
AMP	Nucleotide	Purine Metabolism, Adenine containing	0.72	0.03
adenosine 3',5'-diphosphate	Nucleotide	Purine Metabolism, Adenine containing	0.49	0.00
adenosine	Nucleotide	Purine Metabolism, Adenine containing	0.77	0.00
1-methyladenosine	Nucleotide	Purine Metabolism, Adenine containing	1.35	0.04
guanine	Nucleotide	Purine Metabolism, Guanine containing	0.51	0.01
orotate	Nucleotide	Pyrimidine Metabolism, Orotate containing	1.30	0.00
3-ureidopropionate	Nucleotide	Pyrimidine Metabolism, Uracil containing	0.57	0.05
2'-deoxycytidine	Nucleotide	Pyrimidine Metabolism, Cytidine containing	1.59	0.02
N1-Methyl-2-pyridone-5-carboxamide	Cofactors and Vitamins	Nicotinate and Nicotinamide Metabolism	1.60	0.02
N1-Methyl-4-pyridone-3-carboxamide	Cofactors and Vitamins	Nicotinate and Nicotinamide Metabolism	1.65	0.00
dehydroascorbate	Cofactors and Vitamins	Ascorbate and Aldarate Metabolism	1.31	0.02
catechol sulfate	Xenobiotics	Benzoate Metabolism	0.53	0.00
ergothioneine	Xenobiotics	Food Component/Plant	0.65	0.07
salicylate	Xenobiotics	Drug - Topical Agents	0.48	0.01
O-sulfo-L-tyrosine	Xenobiotics	Chemical	1.60	0.02

Supplementary Table 2. Virus and mouse line assignments for the chemogenetic experiments. Related to Methods.

Experiment	Mouse line	Virus
Chemogenetic activation of anterior thalamic reticular nucleus (aTRN)	GAD2^{cre}	AAV-hSyn-DIO-hM3Dq-mCherry
		Control virus: AAV-hSyn-DIO-mCherry
Chemogenetic inhibition of aTRN	GAD2^{cre}	AAV-hSyn-dF-HA-KORD-IRES-mCitrine
		Control virus: AAV-hSyn-DIO-mCherry
Chemogenetic activation of aTRN following microglia depletion	CX3CR1$^{CreERT2/+}$:R26$^{iDTR/+}$	AAV-hSyn-HA-hM3Dq-mCherry
		Control virus: AAV-CAG-GFP
