# Supplementary material for: Microglia modulate stable wakefulness via the thalamic reticular nucleus in mice
Source: Nat Commun. 2021 Jul 30;12:4646. doi: 10.1038/s41467-021-24915-x (PMC8324895; doi:10.1038/s41467-021-24915-x)
Supplement: Supplementary file 3 — Reporting Summary [file 41467_2021_24915_MOESM3_ESM.pdf]

nature portfolio

Corresponding author(s): Qiaojie Xiong
Last updated by author(s): Jun 29, 2021

Reporting Summary

Nature Portfolio wishes to improve the reproducibility of the work that we publish. This form provides structure for consistency and transparency in reporting. For further information on Nature Portfolio policies, see our Editorial Policies and the Editorial Policy Checklist.

Statistics

For all statistical analyses, confirm that the following items are present in the figure legend, table legend, main text, or Methods section.

n/a	Confirmed	
☐	☒	The exact sample size (n) for each experimental group/condition, given as a discrete number and unit of measurement
☐	☒	A statement on whether measurements were taken from distinct samples or whether the same sample was measured repeatedly
☐	☒	The statistical test(s) used AND whether they are one- or two-sided<br>*Only common tests should be described solely by name; describe more complex techniques in the Methods section.*
☐	☒	A description of all covariates tested
☐	☒	A description of any assumptions or corrections, such as tests of normality and adjustment for multiple comparisons
☐	☒	A full description of the statistical parameters including central tendency (e.g. means) or other basic estimates (e.g. regression coefficient) AND variation (e.g. standard deviation) or associated estimates of uncertainty (e.g. confidence intervals)
☐	☒	For null hypothesis testing, the test statistic (e.g. F, t, r) with confidence intervals, effect sizes, degrees of freedom and P value noted<br>*Give P values as exact values whenever suitable.*
☒	☐	For Bayesian analysis, information on the choice of priors and Markov chain Monte Carlo settings
☒	☐	For hierarchical and complex designs, identification of the appropriate level for tests and full reporting of outcomes
☐	☒	Estimates of effect sizes (e.g. Cohen's d, Pearson's r), indicating how they were calculated

Our web collection on statistics for biologists contains articles on many of the points above.

Software and code

Policy information about availability of computer code

Data collection	Sirena acquisition 1.7.5 (Pinnacle Technology), a commercial available software for collection of data in mice with EEG/EMG electrodes implantation.<br>Cheatah data acquisition 5.6.3 (Neuralynx), a commercial available software for collection of data in mice with EEG/ EMG electrodes and tetrode implantation.<br>PatchMaster V2X53 (HEKA Electronics), a commercial available software for collection of in vitro patch recording data.<br>FV10-ASW 04.02.02.09 (Olympus), a commercial available software for confocal image collection.<br>Ethovision XT (Noldus), a commercial available software for tracking and measurement of locomotor activity.
Data analysis	Sirena sleep 1.7.5 (Pinnacle Technology), a commercial available software for processing of EEG and EMG signals.<br>MClust 4.0, an open source Matlab code used for isolation of single units collected during tetrode recording.<br>BrainMesh, an open source Matlab code used for 3D reconstruction of mouse brain.<br>Matlab 2020a, a commercial available software for statistical analysis.<br>MetaboAnalyst 4.0, a web based tool for metabolic profiling<br>Clampfit 10.0 (Axon), for analysis of patch recording data.<br>ImageJ 1.50i (NIH), an open source software for processing of confocal images.<br>NeuronJ, a plugin available in ImageJ, for measurement of microglia morphology.

For manuscripts utilizing custom algorithms or software that are central to the research but not yet described in published literature, software must be made available to editors and reviewers. We strongly encourage code deposition in a community repository (e.g. GitHub). See the Nature Portfolio guidelines for submitting code & software for further information.

Data

Policy information about availability of data

All manuscripts must include a data availability statement. This statement should provide the following information, where applicable:

- Accession codes, unique identifiers, or web links for publicly available datasets
- A description of any restrictions on data availability
- For clinical datasets or third party data, please ensure that the statement adheres to our policy

The data that support the findings of this study are available in source data. Other data are available upon reasonable request. Mouse brain atlas is available on Allen Institution (https://mouse.brain-map.org/experiment/thumbnails/100048576?image_type=atlas). Source data are provided with this paper.

Field-specific reporting

Please select the one below that is the best fit for your research. If you are not sure, read the appropriate sections before making your selection.

☒ Life sciences ☐ Behavioural & social sciences ☐ Ecological, evolutionary & environmental sciences

For a reference copy of the document with all sections, see nature.com/documents/nr-reporting-summary-flat.pdf

Life sciences study design

All studies must disclose on these points even when the disclosure is negative.

Sample size	Sample size was determined according to published results using similar approaches.<br>https://www.nature.com/articles/s41467-019-08350-7<br>https://www.cell.com/fulltext/S0092-8674(13)01481-5
Data exclusions	We excluded the data from the animals with off-target viral injection or tetrode implantation, and animals that did not complete the recordings.
Replication	Experiments were repeated at least three times to ensure reproducibility, and at least three animals from each group were included in the analysis. The included animals were selected based on their completion of the experiments, the quality of recordings, and the correct locations of viral infection validated post hoc. All attempts at replication were successful.
Randomization	The animals were allocated into all experimental groups were both age and sex matched.
Blinding	The investigators who performed animal treatments and recordings were blinded for data analyses.

Reporting for specific materials, systems and methods

We require information from authors about some types of materials, experimental systems and methods used in many studies. Here, indicate whether each material, system or method listed is relevant to your study. If you are not sure if a list item applies to your research, read the appropriate section before selecting a response.

Materials & experimental systems

n/a	Involved in the study	
☐	☒	Antibodies
☒	☐	Eukaryotic cell lines
☒	☐	Palaeontology and archaeology
☐	☒	Animals and other organisms
☒	☐	Human research participants
☒	☐	Clinical data
☒	☐	Dual use research of concern

Methods

n/a	Involved in the study	
☒	☐	ChIP-seq
☒	☐	Flow cytometry
☒	☐	MRI-based neuroimaging

Antibodies

Antibodies used	Primary antibody: rabbit anti-Iba1 (1:1000, 019-19741, Wako), goat anti-GFP (1:1000, 600-101-215, Rockland), chicken anti-GFAP (1:1000, ab-4674, Abcam), mouse anti-GAD67 (1:500, clone 1G10.2, MAB5406, Millipore), mouse anti-ceramide (1:50, MID-15B4, Enzo), rabbit anti-PV (1:1000, ab-11427, Abcam) and rabbit anti-NeuN (1:1000, ab-177487, Abcam).<br>Secondary antibody: donkey anti-goat 488 (1:1000, A-11055, ThermoFisher), donkey anti-mouse 594 (1:1000, A-21203, ThermoFisher), donkey anti-mouse 647 (1:1000, A32787, ThermoFisher), donkey anti-rabbit 488 (1:1000, 711-545-152, JacksonImmunoResearch), donkey anti-chicken (703-585-155, JacksonImmunoResearch), and donkey anti-rabbit 594 (1:1000, 711-585-152, JacksonImmunoResearch).

Validation	All antibodies have been validated in previously literatures and manufacture's website. The validation for primary antibody in literatures shown as following:<br>rabbit anti-Iba1; Species, mouse; Application, IF; Science 367:688-694 (2020)<br>goat anti-GFP; Species, mouse; Application, IF; Nature 428:668-673 (2004)<br>chicken anti-GFAP, Species, mouse; Application, IF; Nat. Commun. 11:2138 (2020)<br>mouse anti-GAD67; Species, mouse; Application, IF; Nature 489:150–154 (2012)<br>rabbit anti-PV; Species, mouse; Application, IF; Neuron, 95(2):424-435.e6 (2017)<br>mouse anti-ceramide; Species, mouse; Application, IF; J Lipid Res 48:968-975 (2007)<br>rabbit anti-NeuN; Species, mouse; Application, IF; Sci. Rep.,9:3706 (2019)

Animals and other organisms

Policy information about studies involving animals; ARRIVE guidelines recommended for reporting animal research

Laboratory animals	Male and female adult (2–5 months old) wild-type C57BL/6J, CX3CR1CreERT2/+, CX3CR1CreERT2/+:R26iDTR/+, CX3CR1CreERT2/+:R26iDTR/-, GAD2Cre mice and Acer3-/- mice were used for the experiments. Mice were entrained to an environment with 12-h/12-h light/dark cycle with 64-79F and 30-70% humidity. Food and water available ad libitum.
Wild animals	the study did not involve wild animals
Field-collected samples	the study did not involve samples collected from fields
Ethics oversight	Animal procedures were approved by the Stony Brook University Animal Care and Use Committee and carried out in accordance with National Institutes of Health standards.

Note that full information on the approval of the study protocol must also be provided in the manuscript.
